# Characterization of known protein complexes using k-connectivity and other topological measures

**DOI:** 10.12688/f1000research.2-172.v2

**Published:** 2015-10-09

**Authors:** Suzanne R Gallagher, Debra S Goldberg

**Affiliations:** 1 Department of Computer Science, University of Colorado, Boulder CO, 80302, USA

**Keywords:** Protein folding, Complex size, Protein-protein interaction, Saccharomyces cerevisiae, X-ray crystallography

## Abstract

Many protein complexes are densely packed, so proteins within complexes often interact with several other proteins in the complex. Steric constraints prevent most proteins from simultaneously binding more than a handful of other proteins, regardless of the number of proteins in the complex. Because of this, as complex size increases, several measures of the complex decrease within protein-protein interaction networks. However, k-connectivity, the number of vertices or edges that need to be removed in order to disconnect a graph, may be consistently high for protein complexes. The property of k-connectivity has been little used previously in the investigation of protein-protein interactions. To understand the discriminative power of k-connectivity and other topological measures for identifying unknown protein complexes, we characterized these properties in known Saccharomyces cerevisiae protein complexes in networks generated both from highly accurate X-ray crystallography experiments which give an accurate model of each complex, and also as the complexes appear in high-throughput yeast 2-hybrid studies in which new complexes may be discovered. We also computed these properties for appropriate random subgraphs.We found that clustering coefficient, mutual clustering coefficient, and k-connectivity are better indicators of known protein complexes than edge density, degree, or betweenness. This suggests new directions for future protein complex-finding algorithms.

## Background

Proteins are a critical unit in biology. Rather than performing their function alone, many proteins form *protein complexes*, groups of proteins that bind together to perform a specific task. Some of these complexes, such as the proteasome, are well-characterized, but others are not. In addition, it is hypothesized that there are many protein complexes in the cell that have not yet been identified. Complexes play an important role in the function of the cell, and by discovering new complexes and learning more about their composition and structure, we can gain insights into cellular biology.

Ever since high-throughput protein-protein interaction data sets have become widely available, scientists have used the interaction data to create graphs called protein-protein interaction (PPI) networks. The vertices in PPI networks represent proteins, and there is an edge between two vertices if the corresponding proteins interact. These graphs are not perfect models of protein interaction in an organism since the experiments that produced the edges are error-prone and contain both false positives and false negatives. Despite these errors, however, they are useful tools for studying the proteome of an organism.

One use for PPI networks is to predict unknown protein complexes from the interaction data. Previous algorithms have used several different properties to find complexes. By far the most common property has been edge density, the fraction of pairs of nodes (possible edges) that have an edge connecting them ^1– 7^. Most edge density algorithms search for subgraphs with edge density above a certain threshold ^1– 4^. Other properties that have been used include clustering coefficient ^7^, degree statistics ^7, 8^, maximum flow ^9^, and path length ^10, 11^. Biological networks have also been examined using the properties of mutual clustering coefficient ^12, 13^ and betweenness centrality ^14– 17^.

The *k*-connectivity of a graph is a measure of how many distinct paths exist between any pair of vertices. A graph or subgraph is *k-connected* if there are *k* disjoint paths between every pair of nodes, or equivalently, if the removal of at least *k* vertices or edges from the graph are required in order to disconnect it. We believe that a high *k*-connectivity may be more indicative of a protein complex than other measures, and can serve to identify protein complexes even with low edge density. If each protein in the complex binds to some number of adjacent proteins, then as the number of proteins in the complex increases, the edge density will decrease because the maximum number of proteins that a single protein can bind to is limited by steric constraints. The *k*-connectivity, however, will stay roughly constant as long as each protein remains bound to roughly the same number of neighbors. Also, *k*-connectivity implies a certain degree of stability, and a complex with a high *k*-connectivity might be able to retain its structure and even partial function in the event of a mutation that caused an interaction to be lost or for a certain protein to be missing altogether.

*k*-connectivity has only rarely been used in connection with finding protein complexes. Habibi *et al.* ^18^ found that, in mass spectrometry data, *k*-connectivity was a better indicator of protein complexes than edge density. Hartuv and Shamir ^19^ looked for connected subgraphs of n proteins that are *n*/2-connected; however, because their stopping condition is a function of the number of proteins in the subgraph, this is closer to a measure of edge density than *k*-connectivity.

In order to test the hypothesis that *k*-connectivity is a useful indicator of complexes in pairwise interaction data, we examined known complexes in the iPFam ^20^ and MIPS ^21^ databases. For each of these known complexes, we computed *k*-connectivity as well as various other topological properties, with a particular focus on those used in previous complex-finding algorithms: edge density, degree statistics, clustering coefficient, mutual clustering coefficient, number of triangles and 4-cycles, and betweenness centrality. We calculated these statistics in protein interaction graphs representing complexes. For each complex we used interactions determined by low-throughput X-ray crystallography data, where available, as well as high throughput yeast 2-hybrid (Y2H) studies. Finally, in addition to surveying these topological measures in complexes, we compared them to those of random complex-like subgraphs, which we call *pseudocomplexes*, pulled from the PPI network. This allows us to assess the utility of each of these statistics for discovering unknown protein complexes.

Our study compliments the Habibi *et al.* ^18^ study in several ways. First, we used low throughput X-ray crystallography for data on complexes where it was available in order to obtain a “ground truth” about interactions in complexes. The information from this ground truth data, while only available on a limited number of complexes, can give evidence that *k*-connected subgraphs are an important property of complexes independent of the data set in which we are looking at them. Second, we used Y2H studies for our high throughput data rather than mass spectrometry studies used by Habibi *et al.* Our use of a different type of high throughput interaction data can offer evidence that the ability of *k*-connectivity to find complexes can be applied more generally rather than being a particular property of mass spectrometry data. Y2H data is also better suited for network studies due to the fact that it is binary; Y2H assays reveal the presence of an interaction between exactly two protein, unlike mass spectrometry studies which involve interactions between large sets of proteins and can be difficult to translate into the binary interactions required by networks ^22^. Third, we examine a wider variety of statistics than the earlier study, which focused exclusively on *k*-connectivity and edge density, which gives a more extensive look at various statistics that could be useful in complex-finding, and how *k*-connectivity and edge density both rank among these. Finally, in addition to surveying these topological measures in complexes, we compared them to those of random complex-like subgraphs, which we call pseudocomplexes, pulled from the PPI network. This allows us to assess the utility of each of these statistics for discovering unknown protein complexes.

## Methods

### Data

We obtained details about protein complexes in *Saccharomyces cerevisiae* from two different sources. The first source was iPFam, where we were able to obtain data about protein complexes as well as which proteins interact within the complex ^20^. These interactions were determined via X-ray crystallography, which, while not perfectly accurate, should be considered highly reliable. Unfortunately, only 13 complexes with at least three distinct proteins were included in this database. The second source of data on known complexes was the MIPS database ^21^. The MIPS database is far more extensive, but only contains the proteins present in the complex, not the interactions that occur within the complex.

We obtained pairwise Y2H interaction data from Biogrid and created an interaction graph using a composite of all Y2H studies in yeast available on Biogrid ^23^. We did not include data from high-throughput affinity purification-mass spectrometry experiments, as did Habibi *et al.* ^18^, because these experiments are biased towards protein complex interactions, and we sought to understand the properties of protein complexes and how these differ from a random background. To discover new protein complexes, it is appropriate to use all available data, as in the Habibi *et al.* study, but this was not our purpose. In addition, we wished to avoid complications from representing mass spectrometry interactions, which are not intrinsically binary, in a binary graph. We used high-throughput Y2H interactions exclusively because they are intrinsically binary, and do not suffer from known biases towards interactions within protein complexes. For similar reasons, we did not use the PCA binary interactions from Tarassov ^24^ because that study used known complexes to filter the results and therefore would be biased in favor of known complexes.

The high-throughput Y2H data set, however, has a high error rate and includes both false positives (proteins that don’t interact but have been reported to interact in one or more studies) and false negatives (proteins that do interact but whose interaction has not been reported in a Y2H study). We considered using the Y2H Union subset of interactions ^25^, a subset of the interactions with higher confidence, but there aren’t enough interactions in this data set between proteins in the same complex to give us meaningful results; only 25 of the 154 complexes in MIPS induced a connected graph, and of those 25, only 4 had more than 3 proteins in the data. This was not enough data to give a meaningful picture of complexes, so we decided it was better to accept the lower quality but higher number of interactions from the composite data set. It is worthwhile to discover metrics that would allow us to find protein complexes in the abundantly available data. We therefore decided to accept a lower specificity and a higher number of false positives in order to increase the sensitivity.

In order to avoid confusion, for the remainder of the paper, we will refer to the entire collection of proteins and interactions determined by Y2H interactions as the “network”. The collection of proteins and interactions in a complex will be a “graph” while a subset of those interactions will be a “subgraph”.

**Figure 1.**
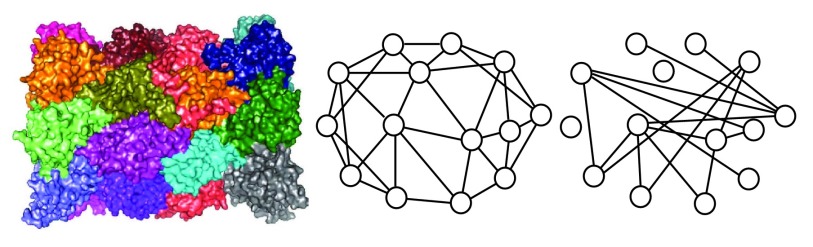
The 20S proteasome and the graphs that represent it. Image on the left is a surface view of the protein. The graph in the middle represents the interactions from the isolated complex (from iPFam), while the graph on the right contains the same proteins but gets its edges from the Y2H network (from Biogrid).

For the complexes from iPFam, we looked at both the interactions determined by the X-ray crystallography on isolated complexes and also the graph induced in the Y2H network by the proteins determined to be in the complex and all Y2H edges amongst these proteins. See Figure 1. The X-ray crystallography data set gives us an idea of how complexes might look in a complete and accurate interaction network, while the Y2H data set gives us an idea of how complexes look in our real error-prone data. For the complexes from MIPS, we were only able to look at the induced graphs from the Y2H data. The code used for calculating the statistics of protein complexes can be found at https://github.com/suzanneg/complex-stats.

### Graph properties

We assessed the following graph measures:

***Edge and vertex *k*-connectivity.*** Measures of the number of distinct paths between any pair of vertices. A graph or subgraph is *k-edge-connected* ( *k-vertex-connected*) if between every pair of nodes there are at least *k* edge-disjoint (intermediate vertex disjoint) paths. Equivalently, any *k*-1 edges (vertices) can be removed from the graph without disconnecting it. In the remainder of this paper, *k*-connectivity refers to vertex *k*-connectivity.

***Edge density.*** The number of interactions (edges) divided by the number of possible interactions (pairs of vertices).

***Degree statistics.*** The maximum, minimum, and mean degrees for each graph, along with the standard deviation of the mean. In order to compare these statistics between complexes with differing numbers of proteins, we normalize by dividing the degree statistics by the number of vertices in the graph.

***Clustering Coefficient (CC).*** A measure of how many of a vertex’s neighbors are neighbors of each other. Over a graph or subgraph, clustering coefficient is defined as 3 times the number of triangles divided by the number of length 2 paths.

***Mutual Clustering Coefficient (MCC).*** For a pair of vertices, the percentage of their neighbors that they share. There are several different ways of defining the mutual clustering coefficient between two vertices, but for our purposes, we define it as the number of shared neighbors divided by the minimum degree (number of neighbors) of the two vertices. This method was the best of the ratio methods from Goldberg and Roth ^13^ for assessing confidence in PPI networks. We calculate the MCC between all pairs of vertices in a complex, and as with degree, we report the maximum, minimum, mean, and standard deviation.

***Motifs.*** Particular subgraphs in each complex. We were interested in the number of triangles and 4-cycles.

***Betweenness centrality.*** For a vertex, the number of shortest paths between all other pairs of vertices that contain that vertex. Again, we report the maximum, minimum, mean, and standard deviation. As with the degree statistics, we normalize by dividing by the number of vertices in the graph. Because complexes are expected to be well-connected, we expect betweenness values to be small.

### Subgraphs

For each graph of a complex, we looked at three subgraphs: 1) the original graph, which includes vertices representing all proteins in the complex; 2) a “haircut” subgraph, where we recursively eliminate all vertices of degree 1 or less, ensuring the subgraph has a minimum degree of 2 (this is the same as the haircut part of the algorithm of Bader and Hogue ^7^); and 3) the subgraph that is *k*-connected for the highest value of *k*, which we call the most highly connected subgraph (MHCS).

We look at these additional subgraphs because we believe that several properties will be more discernible in these sub-graphs, so that these subgraphs are more likely to be able to be discovered by a complex-finding algorithm. The single vertices eliminated by the haircut are unlikely to be discovered by any complex-finding algorithm, and including them lowers the edge density, clustering coefficient, and *k*-connectivity of the graph, as well as raising the betweenness of the adjacent vertex. The MHCS clearly highlights *k*-connectivity, but many other properties are also higher in the MHCS than in the original graph.

### Assessment

In order to assess the significance of properties in the complexes and the Y2H network as a whole, we used two different methods of generating random graphs. For the Y2H network, we generated networks with the same number of vertices and the same edge distribution by “switching”. Switching works by choosing two random edges with different endpoints, ( *A*, *B*) and ( *C*, *D*), removing those edges, and replacing them with edges ( *A*, *D*) and ( *C*, *B*). We use the method recommended by Milo *et al.* ^26^: for a network with *n* vertices, the process is repeated 100 *n* times to ensure proper mixing. The end result is a random network with the same degree distribution as the original network ^27^. This process is repeated 10 times, giving us 10 random networks for comparison.

A somewhat different method was used to assess the significance of the properties of the complexes. Switching would only allow us to compare a protein complex graph with another graph of the same degree distribution, when what we really want is to compare it to other graphs from the Y2H network. Our question is “how likely are we to see this result in the actual network where there is not a complex?” so we seek graphs that are similar to our complexes. For each complex with at least 4 proteins, we found a “matched” graph that we call a *pseudocomplex*. A pseudocomplex *P* that matches a complex with n proteins is generated by taking an edge from a random triangle from the Y2H network and letting *P _2_* = this edge and the two nodes it connects. For *i* > 2, we generate P _*i*_ from P _*i-1*_ by taking a random edge in the Y2H network attached to P _*i-1*_ and adding the vertex at the other end and all edges from this vertex to P _*i*- *1*_. Repeat this process until we have the same number of vertices as the original complex and let P = P _*n*_. We chose a random edge rather than a random neighbor so that nodes connected by multiple edges would be more likely to be chosen, making the final graph more “complex-like.” We started with an edge from a triangle rather than a random edge for the same reason, because most (though not all) complexes contained at least one triangle. Although this bias may make pseudocomplexes more likely to contain a triangle than real complexes are, we believed it was better to be overly conservative in this respect. We considered only complexes with at least 4 proteins because fewer nodes in a connected subgraph require some measures to be unreasonably high, and this would skew our comparisons. We calculated the same measures for pseudocomplexes as we did for the complex graphs, and compared our results with the real complexes.

## Results

### Results on iPFam complexes

**Figure S1.**
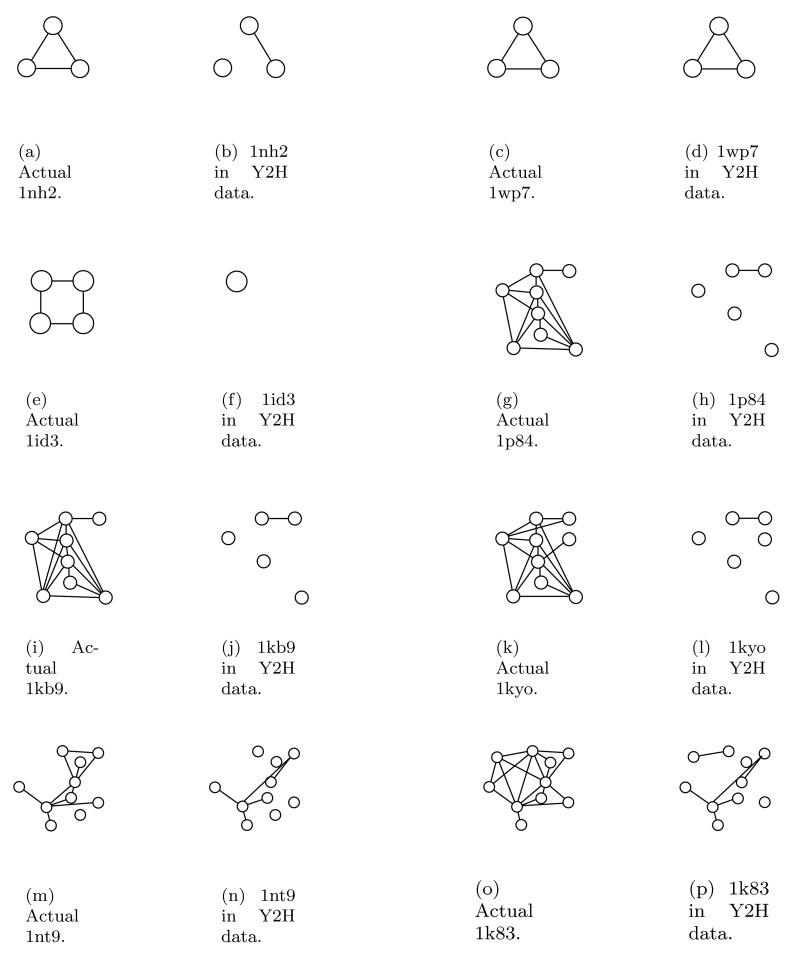
Complexes from iPFam, and those same proteins in Y2H data. IDs are from the RCSB Protein Data Bank.

**Figure S2.**
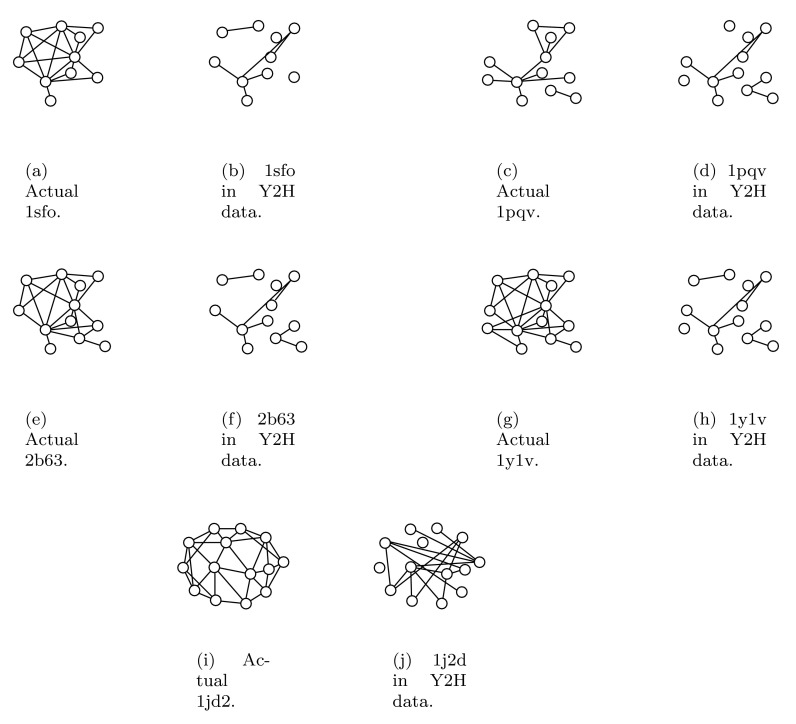
More complexes from iPFam and the same proteins in Y2H data. IDs are from the RCSB Protein Data Bank.

There were 35 studies in iPFam that involved complexes with at least 3 proteins. Some of these studies were of the same or similar complexes; we grouped studies together if they produced the exact same graph, i.e. the same proteins with the same set of interactions. This grouping gave us 13 distinct graphs. All graphs are illustrated in Figure S1 and Figure S2 along with the subgraphs they induced in the Y2H data. In some cases, it is possible that two different studies of the same complex may have produced different graphs, but we will treat all distinct graphs as separate entities. Full statistics for the complexes from iPFam are in the Supplementary material; because we had interaction data from X-ray crystallography, we were able to analyze a reliable graph representation for these complexes.

In all except two cases, the interactions from the X-ray crystallography produced connected graphs. Most complexes were only 1-connected due to the presence of a small number of degree 1 vertices; in all cases except one, the haircut subgraphs were at least 2-connected. About half the complexes had a subgraph that was at least 3-connected. In general, the edge density could be closely correlated with the number of vertices in the complex; complexes with only 3 proteins produced cliques while those with 12 or more tended to have edge densities closer to 1/3. Clustering coefficients had a similar pattern to edge density in that the value was closely correlated with the number of vertices in the complex. Mutual clustering coefficients were more scattered, but also tended to decrease as the number of vertices increased.

When we look at the iPFam complexes in the Y2H data, we see that 9 of the 13 have all of their proteins present, 3 have slightly more than 60 percent, and 1 has only 1 out of 4 proteins present. Only in one, a complex with 3 proteins, were all of the interactions from the X-ray crystallography present in the Y2H data. With the exception of that complex, none of the complexes induced connected graphs, and they all had edge densities of less than 0.1. In all except two cases, the haircut produced an empty subgraph. Only two complexes had a subgraph that was at least 2-connected. Most graphs had clustering coefficients of 0. Average mutual clustering coefficients were higher, between 0.14 and 0.63. Comparing these results with the results obtained using the X-ray crystallography data set gives an indication of how many interactions have not been detected using a Y2H assay and how these false negatives make it difficult to detect complexes. Note that, in Figure 1, only 8 of the 32 edges in the “correct” graph generated by X-ray crystallography were also observed in the high-throughput graph generated by Y2H experiments.

### Results on MIPS complexes

**Figure 2.**
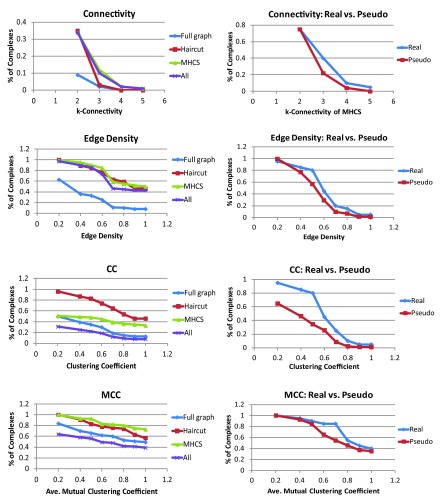
Results on *k*-connectivity, edge density, clustering coefficient, and mutual clustering coefficient. For each statistic, the graph on the left contains the percent of complex graphs, haircut graphs, MHCS, and all connected components that are above a given threshold. The graph on the right contains percentages of real complexes and pseudocomplexes that are above the threshold. Note that only complexes that had some interactions between their component proteins are included in these graphs.

**Figure 3.**
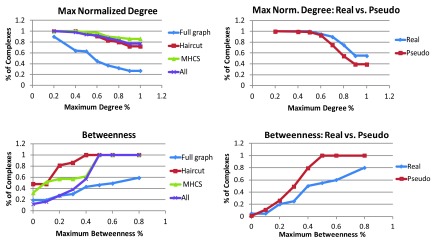
Results on degree and betweenness. For maximum normalized degree, the graph on the left contains the percent of complex graphs, haircut graphs, MHCS, and all connected components that are above a given threshold. The graph on the right contains percentages of real complexes and pseudocomplexes that are above the threshold. For maximum betweenness, the graphs show the percent of complexes below a threshold. Note that only complexes that had some interactions between their component proteins are included in these graphs.

Results on *k*-connectivity, edge density, clustering coefficient, and mutual clustering coefficient are summarized in Figure 2, and results for normalized maximum degree and betweenness centrality are summarized in Figure 3. The left-hand graphs contain full results on the complexes, while the right-hand graphs contain comparisons with pseudocomplexes. Note that because at least 4 proteins are needed to create a pseudocomplex, the real complexes on the right-hand side are a subset of the complexes on the left-hand side. Full results are contained in the Data Files.

Full results of MIPS protein complex topological survey Full results from our survey of MIPS database complexes including all of the statistics. There is a separate excel sheet for results for the full graph (Normal), the haircut subgraph (Haircut), and the most highly connected subgraph (Max Connect). The final sheet (No Interaction) contains a list of all complexes where there were no interactions between any of the component proteins in the Y2H data. In each spreadsheet, complexes that induced a connected subgraph and complexes that did not induce a connected subgraph are listed separately. Further instructions for how to interpret these tables are in the README spreadsheet. Click here for additional data file. Copyright: © 2015 Gallagher SR and Goldberg DS 2015 Data associated with the article are available under the terms of the Creative Commons Zero "No rights reserved" data waiver (CC0 1.0 Public domain dedication).

The results on *k*-connectivity are shown at the top of Figure 2. The graph on the top left gives results on *k-*connectivity in the full complex graphs, the haircut graphs, the MHCS, and all connected components of complexes. From this we can observe that most complexes are at most 1-connected, but when degree 1 vertices are removed, all complexes not destroyed by this operation (39% of the total) are 2-connected. Many complexes also had a subgraph with even higher connectivity.

Comparisons between *k*-connectivity in real complexes and pseudocomplexes are shown on the top right of Figure 2. Note that this graph, unlike the other graphs comparing complexes and pseudocomplexes, gives the *k*-connectivity of the MHCS rather than the entire complex or pseudocomplex. This was done because most complexes and pseudocomplexes had a *k*-connectivity of 1. It was only looking at the MHCS that the differences between complexes and pseudocomplexes became apparent. While roughly the same number of complexes and pseudocomplexes had a 2-connected subgraph, a far higher percentage of complexes had more highly connected subgraphs. Note that pseudocomplexes were designed to have, with high probability, a triangle (a 2-connected subgraph).

The remainder of Figure 2 summarizes the results on edge density, clustering coefficient, and mutual clustering coefficient. From the raw edge density values, we can see that the edge density of most complexes is nowhere near as high as it would be if complexes were cliques or near-cliques: only about 1 in 10 complexes had an edge density above 0.7. In the comparisons with pseudocomplexes, we see that the edge density of complexes and pseudocomplexes is fairly similar, with the density of complexes being slightly higher. The difference is less dramatic, however, than it is for *k*-connectivity due to the high standard deviation of the pseudocomplexes: the point where the maximum difference between known complexes and pseudocomplexes can be seen (the obvious cut-off point between real complexes and pseudocomplexes), 0.5, was well within a standard deviation of the average for pseudocomplexes. The obvious cut-off point for *k*-connectivity, 3-connected, by contrast was more than a standard deviation away from the average of the pseudocomplexes. The number of complexes with high clustering coefficients was also quite small, but clustering coefficients had a far more dramatic contrast with pseudocomplexes, especially for lower thresholds. Again, however, there was a fairly high deviation among pseudocomplexes. Mutual clustering coefficients have higher raw values but much less of a contrast with pseudocomplexes. When the deviation of pseudocomplexes is considered, mutual clustering coefficient does not differentiate from complexes as well as *k*-connectivity.

There are a few further things to note about clustering coefficients and mutual clustering coefficients. Clustering coefficients were quite high in haircut graphs, but this is somewhat misleading. The haircut can remove length 2 paths from the graph but cannot remove any triangles; therefore, we would expect to increase clustering coefficient, but this increase would not necessarily help us in finding complexes. Average mutual clustering coefficient is much higher than clustering coefficient. The reason for this is that there are many more 4-cycles than triangles. While triangles are overrepresented in the Y2H network as compared to a random network of the same degree distribution produced by switching (4681 v. 1609.8, 2.9 times as many), 4-cycles are also overrepresented (98166 v. 24045.0, 4.1 times as many). The frequencies of triangles and 4-cycles relative to random networks has been calculated for a previous yeast PPI network, also with the result that both were overrepresented, with 4-cycles also overrepresented by a higher margin, though this was not stated explicitly ^28^. This pattern does not, however, appear to hold completely true for all PPI networks; specifically, in *Drosophila melanogaster*, triangles appear to be more overrepresented than 4-cycles ^29^.

This pattern also seems to hold in the complex graphs. Neither triangles nor 4-cycles were particularly prevalent in complexes relative to pseudocomplexes (which were each seeded within a triangle), but 4-cycles were more prevalent than triangles. In 50% of complexes, there were more 4-cycles as compared to matching pseudocomplexes. However, only 29% of complexes had more triangles than their matching pseudocomplexes.

The normalized results for maximum degree and comparisons with pseudocomplexes are in Figure 3. In many of the complexes we looked at, there was at least one protein of high degree that had an interaction with all or almost all of the other proteins in the complex, forming a “star” or a “hub and spoke” in the graph. This has been previously suggested by Bader and Hogue as a way to model the interactions in complexes that were found experimentally using affinity-purification ^8^. However, there are some problems with using this idea to search for complexes in the data. The first is that we did not notice a strong correlation between proteins with high degree and proteins that appear in known complexes; roughly 30% of proteins of degree 3 or higher in our data set appeared in at least one complex, and this number remained roughly constant as we increased the degree threshold until it eventually started decreasing due to the limited number of proteins with degrees above 20. The second problem is that if we look at the protein in a complex with the most interactions with other proteins in that complex, the majority of its interactions in the Y2H data are not within the complex. Therefore, the strategy of looking for a protein of high degree and taking it and all of its neighbors as a complex seems unlikely to produce meaningful results for finding protein complexes in Y2H data.

Normalized maximum betweenness is also shown in Figure 3. Note that for the panels for maximum betweenness, unlike the others, we report the number of complexes that were less than a given threshold rather than greater than the threshold. Some graphs did not have enough vertices (at least 3 in a connected component) to make a valid measure of betweenness; these were not included in the statistics. Betweenness statistics are not given for unconnected complexes because not all pairs of vertices have paths between them. Traditionally, betweenness has been used as a way to divide the PPI network into functional modules by identifying edges with high betweenness as edges between distinct modules or complexes, so it may seem odd that we are looking at betweenness within a complex. We expect betweenness values to be low, since we expect there to be few if any “bottleneck nodes” in the complex that many shortest paths must go through. Although the minimum betweenness was almost always 0, and average betweenness was relatively small, the maximum betweenness varied quite widely, and there were some vertices with very high normalized betweenness. Surprisingly, the maximum betweenness tended to be higher in the real complexes than in the pseudocomplexes.

## Discussion

### Data

We used a PPI network whose interactions were determined solely by high-throughput Y2H assays. Other binary interaction data sets, such as small-scale experimental data and literature curated interactions, were not used in this study due to the fear that they would be biased in favor of interactions in known complexes. While these interactions would be included in the data set used by an algorithm looking for unknown complexes, they should not be included in an attempt to learn the properties of complexes and what differentiates them from random.

Similarly, we chose not to use non-binary data such as affinity purification data in this study. While these data again might be used in a complex-finding algorithm, the correct way to translate the data from these non-binary experiments into the binary interactions required by graphs is not completely obvious. The two commonly used methods (clique and spoke) produce very different topological properties, and neither captures well the underlying biology. Therefore, we decided to sidestep the issue by using only binary data. Future studies may include finding a way to use these data.

As we carried out this analysis, we were always aware of the fact that our data are error prone. We must keep in mind that the absence of an edge does not mean that there is no interaction. In order to see that we have false negatives, we need only look at the complexes with their interactions determined by X-ray crystallography and compare them to the interactions of those same proteins in the Y2H data ( Figure 1 and Figure S1 and Figure S2). Presumably, if all “real” interactions had been detected, all of the interactions that we see in the X-ray crystallography studies would be present. False positives are a more difficult matter to detect. Again, if we compare the X-ray crystallography to the Y2H data, we see edges in the Y2H graph that weren’t in the X-ray crystallography. However, we cannot simply declare these false positives. It is possible that they truly are false positives. It is also possible that while “false” these interactions are significant due to the fact that they appear in the same complex (e.g. we are incorrectly labeling as a neighbor what should actually be the neighbor of a neighbor). Finally, it is possible that these are true interactions that simply do not appear as part of this complex. A recent study suggests that there are many such binary interactions and that the false positive rate for Y2H data is actually much lower than previously believed ^30^.

While false positives may cause problems in complex-finding algorithms, our survey suggests that false positives may be less of a problem than false negatives. If we had used a cleaner data set, we would have had fewer false positives but also fewer true positives, and we would have had even more difficulty discerning complexes. Even in the data set we used, complexes often did not stand out when compared to pseudocomplexes.

While the errors in the Y2H data are noteworthy, we do not feel that they represent a weakness in our study. To the contrary, a complex-finding algorithm would also be working in this same error-prone data. While it would be interesting to know how a complex would appear in a completely correct network, it is more useful to know how it appears in the data we have.

Another point about our data worth noting involves the pseudocomplexes used for comparison to represent “background” areas of the graph. Because the generating algorithm was trying to find “complex-like” subgraphs, some of our “pseudocomplexes” may in fact be unknown protein complexes. This would skew our results somewhat, but generally gives a conservative comparison; some unique features of true complexes may not be discovered, but it is less likely that noted differences between true complexes and the set of “pseudocomplexes” are spurious.

### Topological measures

We found that edge density may have been overrated as a property of complexes. We found that in Y2H data, the complexes were not particularly clique-like and edge densities were nowhere near as high as most complex-finding algorithms assumed. For example, the algorithm used by King *et al.* ^31^ looks for complexes with an edge density of at least 0.7 with a minimum number of proteins. If this algorithm were applied to Y2H binary interaction data (the data King *et al.* used included multiple types of interactions, some of which were not binary), our research suggests that such a technique would find all of the proteins involved in a complex for just over a tenth of known complexes with 3 or more distinct proteins. An edge density threshold of 0.7 would find the MHCS of about 60% of known complexes, thus finding at least part of the complex, but this still leaves more than a third of complexes undetected. Also, on average, the edge densities in complexes were only slightly higher than the edge densities in the pseudocomplexes, which suggests that edge density may produce many false positives as well. Therefore, while edge density has a role in complex-finding algorithms, we would be skeptical of methods that purport to find complexes in Y2H data based solely on edge density.

Clustering coefficient has not been as popular a parameter for complex-finding algorithms as edge density, but it has long been one of the standard tools used to study the PPI network and its subgraphs. We found that clustering coefficients in real complexes were higher than those from equivalent pseudocomplexes.

Mutual clustering coefficient is another statistic that has not been used extensively in complex-finding algorithms, but we believe shows promise. Many complexes have high average mutual clustering coefficients as seen in Figure 2, and pseudocomplexes often have lower mutual clustering coefficients. An additional reason to believe that mutual clustering coefficient may perform well in a complex-finding algorithm is that mutual clustering coefficient considers 4-cycles as well as triangles in its calculation. As mentioned in the results section, we have found that 4-cycles are overrepresented in the Y2H network as a whole, and seem to be even more overrepresented in complexes. Both clustering coefficient and mutual clustering coefficient seem to have a correlation with complexes and would likely have a role in a new complex-finding algorithm.

Looking at maximum degree, we can see that many complexes have at least one protein with interactions with a high percentage of the other proteins in the complex. At the high end, this differentiated complexes from pseudocomplexes. However, we were not able to correlate proteins of high degree with proteins present in known complexes. Also, even among high-degree proteins that were present in complexes, the majority of the neighbors of those proteins were not co-complexed. For these reasons, we are hesitant to recommend degree as an important part of a complex finding algorithm.

Betweenness was one of the statistics that performed the most unexpectedly. Vertices of high betweeenness are usually believed to be vertices that exist between different biological modules. Under that assumption, we would expect all vertices in a complex to have low betweenness. However, when we looked at complexes under this assumption, we found that most complexes had at least one vertex with a higher betweenness than their pseudocomplex counterparts. Therefore, any algorithm that partitioned the network by looking for high betweenness vertices would run the risk of dividing complexes. It is possible that betweenness could still be used in a complex finding algorithm, but likely not in the way that it has been used traditionally.

The *k*-connectivity of complexes, on the other hand, stood out versus the *k*-connectivities of the pseudocomplexes. Our results were mixed but promising. Most complexes were only 1-connected, but this was due to a small number of degree 1 vertices. When these vertices were removed by the haircut, a 2-connected subgraph usually remained, and many complexes had 3-connected or 4-connected subgraphs. The presence of 3-connected and 4-connected subgraphs is significant; because of the way we generated our pseudocomplexes, they were biased towards including a 2-connected subgraph (the triangle from which the initial edge was selected), but very few had a 3-connected subgraph. Almost none of the pseudocomplexes that were designed to mimic the connected complexes had a 4-connected subgraph.

Another feature that is noteworthy about *k*-connectivity is that, while some of the haircut graphs were empty, none of the others had a *k*-connectivity of 1. Eliminating vertices of degree 1 is not by itself enough to guarantee that a non-empty graph will be at least 2-connected, so this result is significant. It indicates that removing all degree 1 vertices from complexes also eliminates all articulation points, vertices whose removal disconnects the graph, leaving behind a graph where no one vertex can be removed to disconnect the graph. It should also be noted that while our results on *k*-connectivity in the error-prone data were promising, our results in the more accurate X-ray crystallography data were even more so. In the X-ray crystallography data, all complexes had at least a 2-connected subgraph, and the majority of complexes had a 3-connected or 4-connected subgraph. This suggests that as our data become more complete and accurate, highly connected subgraphs will play an even stronger role in searching for complexes.

### The role of *k*-connectivity in future complex-finding algorithms

Our analysis confirms the connection between highly connected subgraphs and protein complexes first suggested by Habibi *et al.* ^18^. The fact that *k-*connectivity was shown to be an important indicator of protein complexes in a different type of experimental data than the one used by Habibi et al suggests that the importance of *k*-connectivity is real and not just an artifact of one type of data.

In their paper, Habibi *et al.* ^18^ present an algorithm for finding complexes based on *k*-connectivity. We are somewhat skeptical of using vertex connectivity alone as the basis of a complex finding algorithm in Y2H data, however, because subgraphs with these connectivities are too common; it is easy to find 2- or 3-connected graphs of almost any size in the PPI network. Starting with a triangle, it is possible by adding one vertex at a time to build a 2-connected subgraph of any size up to 1689 vertices. Starting with a 4-clique, it is possible to build a 3-connected graph of any size up to 913 vertices. Nevertheless, we feel these vertex connectivity results are significant. The MHCS of graphs representing real complexes were much more highly connected than those of pseudocomplexes, despite our method of generating pseudocomplexes being (perhaps unfairly) biased towards higher *k*-connectivity, and less biased towards higher edge density. The presence of a highly connected MHCS was one of the statistics that most differentiated real complexes from pseudocomplexes, suggesting that *k*-connectivity has a role in complex-finding algorithms. The absence of articulation points and the presence of highly connected subgraphs indicates something about the structure of complexes.

We believe *k*-connectivity should be used in conjunction with other properties in a complex-finding algorithm. Several other properties examined in this survey, most notably clustering coefficient and mutual clustering coefficient, were also highly correlated with complexes. A complex-finding algorithm based on these data could try to build a 3- or 4-connected subgraph that also had high clustering coefficients and mutual clustering coefficients. Several existing complex-finding algorithms use multiple criteria, such as MCODE ( *k*-core, clustering coefficient, and edge density) ^7^, the algorithm of King *et al.* (clustering and edge density) ^31^, and the Bayesian network of Qi *et al.* (multiple properties, including edge density, degree statistics, and clustering coefficients) ^32^. Connectivity could also be used to evaluate candidate subgraphs produced by other complex-finding algorithms. Subgraphs found by other methods could be examined to find their most highly connected subgraph, with higher confidence scores being given to those with higher *k*-connectivity values for their most highly connected subgraphs. Finally, we hypothesize that the most highly connected subgraph of a complex graph may correspond to the “core” of a protein-complex as described by Dezso *et al.* ^33^ and Gavin *et al.* ^34^. If true, this would imply that *k*-connectivity could be used in improvements to algorithms that use the core-attachment model ^35, 36^.

## Conclusion

Before designing a new algorithm to find unknown protein complexes in protein interaction data, we must understand the topological properties of known protein complexes. We conducted a principled and comprehensive survey of the topological properties of known protein complexes. We computed vertex *k*-connectivity, edge density, maximum normalized degree, clustering coefficient, mutual clustering coefficient, triangle (3-cycle) count, 4-cycle count, and betweenness centrality in various graphs representing known protein complexes in the high-throughput Y2H data available for new protein complex discovery. For each known protein complex, we computed these properties in the graph induced by proteins contained in the complex in the Y2H network as well as in the haircut and MHCS subgraphs of these, which are more likely to be discoverable by an automated method. To measure the significance of our results we computed the same properties as we did for the complexes on random “complex-like” graphs from the Y2H network.

Although the property of edge density has been the most commonly used measure when searching for complexes in the PPI network, we found that it may not be the best graph measure for protein complex discovery. Instead, we found that *k*-connectivity, clustering coefficient, and mutual clustering coefficient appear to be the most effective measures for differentiating protein complexes from background pseudocomplexes in the pairwise Y2H interaction data. Importantly, our analysis suggests that *k*-connectivity, a graph metric which has rarely been used in the study of protein networks, would improve algorithms designed to find protein complexes in protein-protein interaction data.

## Data availability

The data referenced by this article are under copyright with the following copyright statement: Copyright: © 2015 Gallagher SR and Goldberg DS

Data associated with the article are available under the terms of the Creative Commons Zero "No rights reserved" data waiver (CC0 1.0 Public domain dedication). 

*Figshare:* Characterization of known protein complexes using k-connectivity and other topological measures. doi: 10.6084/m9.figshare.729086 ^37^

## Figures and Tables

**Table S1.** Statistics for iPFam complexes. The number of proteins (n) and interactions (m), edge density (Edge Dens.), maximum degree (Max Degree), clustering coefficient (CC), average mutual clustering coefficient (Ave MCC), average betweenness (Ave Bet.), and the vertex connectivity of the Most Highly Connected Subgraph (MHCS Connect.) for each iPFam complex. The IDs given are from the RCSB Protein Data Bank. X-ray = complex as determined by X-ray crystallography, Y2H = induced subgraph in yeast 2-hybrid data. The number in parentheses in the m Y2H column is the number of interactions from the X-ray crystallography that also occur in the Y2H network. “N/A” means that there were not enough vertices to calculate a given statistic.

	n		m		Edge Dens.		Max Degree	
PDB ID	X-ray	Y2H	X-ray	Y2H	X-ray	Y2H	X-ray	Y2H
1nh2	3	3	3	1(1)	1	0.33	2	1
1w7p	3	3	3	3(3)	1	1	2	2
1id3	4	1	4	0(0)	0.67	N/A	2	0
1p84	8	5	15	1(1)	0.54	0.1	5	1
1kb9	8	5	16	1(1)	0.57	0.1	5	1
1kyo	9	6	17	1(1)	0.47	0.07	6	1
1nt9	10	10	10	5(4)	0.22	0.11	5	4
1k83	10	10	18	6(5)	0.4	0.13	7	4
1sfo	10	10	19	6(5)	0.42	0.13	8	4
1pqv	12	12	11	7(5)	0.17	0.11	6	4
2b63	12	12	22	8(7)	0.33	0.12	8	4
1y1v	13	13	25	8(8)	0.32	0.10	9	4
1jd2	14	14	32	11(8)	0.35	0.12	6	3
	CC		Ave MCC		Ave Bet.		MHCS Connect.	
PDB ID	X-ray	Y2H	X-ray	Y2H	X-ray	Y2H	X-ray	Y2H
1nh2	1	N/A	1	N/A	0	0	2	1
1w7p	1	1	1	1	0	0	2	2
1id3	0	N/A	0.33	N/A	0.5	N/A	2	N/A
1p84	0.61	N/A	0.71	N/A	2	0	3	1
1kb9	0.68	N/A	0.77	N/A	1.75	0	4	1
1kyo	0.554	N/A	0.72	N/A	2.44	0	3	1
1nt9	0.26	0	0.63	0.64	3.5	1.3	2	1
1k83	0.47	0	0.83	0.30	2.9	1.3	3	1
1sfo	0.52	0	0.89	0.30	2.8	1.3	4	1
1pqv	0.13	0	0.37	0.26	4.17	1.17	2	1
2b63	0.45	0	0.71	0.16	4.42	1.17	3	1
1y1v	0.42	0	0.66	0.16	4.85	1.08	3	1
1jd2	0.48	0	0.36	0.14	5.93	3.21	4	1

## Supplementary material

Here we give full results of all statistics on all complexes we studied.

## Results on iPFam complexes

Full results of our survey of iPFam complexes are shown in Table S1. For each of these complexes, we analyzed both the high quality X-ray crystallography data from iPFam as well as the high throughput Y2H data for the proteins in the complex. There were 35 studies in iPFam that involved complexes with at least 3 proteins. Some of these studies were of the same or similar complexes; we grouped studies together if they produced the exact same graph, i.e. the same proteins with the same set of interactions. This grouping gave us 13 distinct graphs. All graphs are illustrated in Figure S1 and Figure S2, along with the subgraphs they induced in the Y2H data. In some cases, it is possible that two different studies of the same complex may have produced different graphs, but we treat all distinct graphs as separate entities.
