# Supplementary material for: Characterization of known protein complexes using k-connectivity and other topological measures
Source: F1000Res. 2015 Oct 9;2:172. Originally published 2013 Aug 13. [Version 2] doi: 10.12688/f1000research.2-172.v2 (PMC4743144; doi:10.12688/f1000research.2-172.v2)
Supplement: Peer review history

# Peer review: Referee response for version 2

**DOI:** 10.5256/f1000research.7362.r11072

**Published:** 2015-12-01

**Authors:** Joel Bader

**Affiliations:** 1 Department of Biomedical Engineering, John Hopkins School of Medicine, MD, USA

This manuscript provides an analysis of different features that could potentially be used as input for methods that identify protein complexes from pairwise interaction data from yeast two-hybrid screens. The manuscript is strong in using X-ray structures as a gold standard for real interactions and in performing parallel analysis of real complexes and pseudo-complexes drawn from reasonably realistic randomized networks. It motivates use of metrics based on clustering and connectivity rather than edge density or centrality.

There are two areas where additional work could increase the value of this manuscript. First would be to examine which metrics perform better for the task of identifying complexes. A reasonable approach would be to use the different features to rank a mixture of real complexes and pseudo-complexes and to see which features perform better at ranking the real complexes above the pseudo-complexes. The second area would be to investigate properties of complexes that affect the performance of different features. I suspect that one of the explanations might be that some methods perform better for smaller vs. larger complexes. It would probably be helpful to look at performance for complexes grouped into size classes.

Suggestions:

In the Background paragraph summarizing other methods, which starts “One use for PPI networks”, I think it would be useful to add “patterns of connectivity as represented by stochastic block models” as a feature used for identifying complexes. I think these are the best-performing methods. Two references are Clauset *et al.*(2008) Park and Bader (2011).

In the Background, the statement “Y2H assays reveal the presence of an interaction between exactly two protein” should be “proteins”. Also, it might be too strong. It is possible that a third protein that forms a complex with the bait and prey and is co-localized to the cell compartment of the Y2H assay could bridge the bait and prey resulting in detection of an indirect interaction. Given access to the x-ray structures, they authors might be able to discover whether any of the published Y2H pairwise interactions are likely to be indirect rather than direct.

In several locations, false positives and false negatives are mentioned. It could be helpful to provide citations to estimates of false-positive and false-negative rates. Examples are Hart *et al. *(2006); Huang *et al.* (2007); Gentleman and Huber (2007); and possibly Cusick *et al.* (2009).

Methods, Assessment: It could be helpful to mention that many of the Y2H assays were asymmetric, with a smaller number of baits used as queries against a larger library of preys. Some randomization methods have attempted to keep the distinction between baits and preys as part of the edge swapping.

Results: Are some of the metrics more sensitive to the size of a complex? Can the authors provide some analysis as a function of protein complex size? Spatial constraints, for example, limit the edge density in real complexes. Clustering coefficients may have a better inherent normalization for cluster size.

Results: It would be helpful to see how the different methods perform in identifying complexes. For example, suppose that the real complexes and pseudo-complexes are grouped together, and then the different features are used to rank-order the mixed collection. The ROC/AUC and PR/F-score could be used to give a rough idea of how well the individual features perform in distinguishing real from randomized complexes.

Figure 2: It would be better to have axes that represent percentages to stop at 100% instead of 120%.

Minor points:

Abstract, “Saccharomyces cerevisiae” should be Italic font.

Abstract, “subgraphs.We found” insert space after period.

Background, “Proteins are a critical unit” change to “Proteins are critical units”

Background, “contain both false positives and false negatives”. Might be better to say “subject to” rather than “contain” because the false negatives aren’t there, not really contained.

Methods, “The code used for calculating the statistics of protein complexes can be found at https://github.com/suzanneg/ complex-stats.” It would also be helpful to provide the code and data sets as supplementary information to ensure continued availability of a snapshot of the material at the time of publication.

I have read this submission. I believe that I have an appropriate level of expertise to confirm that it is of an acceptable scientific standard, however I have significant reservations, as outlined above.

## References

[1] ClausetAMooreCNewmanME: Hierarchical structure and the prediction of missing links in networks. *Nature*.2008;453(7191) : 10.1038/nature06830 98-101 10.1038/nature06830 18451861

[2] ParkYBaderJS: Resolving the structure of interactomes with hierarchical agglomerative clustering. *BMC Bioinformatics*.2011;12 Suppl 1: 10.1186/1471-2105-12-S1-S44 S44 10.1186/1471-2105-12-S1-S44 21342576PMC3044301

[3] HartGTRamaniAKMarcotteEM: How complete are current yeast and human protein-interaction networks?. *Genome Biol*.2006;7(11) : 10.1186/gb-2006-7-11-120 120 10.1186/gb-2006-7-11-120 17147767PMC1794583

[4] HuangHJedynakBMBaderJS: Where have all the interactions gone? Estimating the coverage of two-hybrid protein interaction maps. *PLoS Comput Biol*.2007;3(11) : 10.1371/journal.pcbi.0030214 e214 10.1371/journal.pcbi.0030214 18039026PMC2082503

[5] GentlemanRHuberW: Making the most of high-throughput protein-interaction data. *Genome Biol*.2007;8(10) : 10.1186/gb-2007-8-10-112 112 10.1186/gb-2007-8-10-112 18001486PMC2246275

[6] CusickMEYuHSmolyarAVenkatesanKCarvunisARSimonisNRualJFBorickHBraunPDrezeMVandenhauteJGalliMYazakiJHillDEEckerJRRothFPVidalM: Literature-curated protein interaction datasets. *Nat Methods*.2009;6(1) : 10.1038/nmeth.1284 39-46 10.1038/nmeth.1284 19116613PMC2683745

# Peer review: Referee response for version 1

**DOI:** 10.5256/f1000research.800.r3973

**Published:** 2014-03-05

**Authors:** Nassim Sohaee

**Affiliations:** 1 Department of Mathematics and Information Sciences, University of North Texas, Dallas, TX, USA

This paper is a survey of the topological properties of known protein complexes and is mostly focused on k-connected subgraphs as a good candidate for predicting protein complexes in PPI networks. K-connected subgraphs have been previously proposed as a good alternative for predicting less dense protein complexes. I have the following comments about this paper:

1. The idea of using k-connected subgraphs to predict protein complexes has already been investigated by other researchers. The authors should therefore clearly point out what is new about their concept and how it differs from earlier published methods.
2. According to Sharan *et al. *(2007) the functional similarity of protein pairs will decrease as the distance of the path connecting them increases. Hence, a large k value results in a less functional correlation among proteins. Specifically, for k>= 3, functional similarity significantly decreases and for k<3, or k=1 or 2, the k-connected graph is dense. As this has already been investigated by others the authors need to clearly state what makes their method significant. 

I have read this submission. I believe that I have an appropriate level of expertise to confirm that it is of an acceptable scientific standard, however I have significant reservations, as outlined above.

# Peer review: Referee response for version 1

**DOI:** 10.5256/f1000research.800.r1873

**Published:** 2013-10-24

**Authors:** Lin Gao

**Affiliations:** 1 School of Computer Science and Technology, Xidian University, Shaanxi, China

This is a promising paper. I think the idea is sound, has some novelty, and can potentially improve previous results. The explanations of the figures are of particular note. In addition, the authors could try a larger benchmark complex dataset (CYC2008 category).

I have read this submission. I believe that I have an appropriate level of expertise to confirm that it is of an acceptable scientific standard.
